# Optimizing the endoscopic diagnosis of mediastinal lymphadenopathy: a glimpse on cryobiopsy

**DOI:** 10.1186/s12890-022-02160-2

**Published:** 2022-09-19

**Authors:** Michele Mondoni, Giovanni Sotgiu

**Affiliations:** 1 Respiratory Unit, ASST Santi Paolo E Carlo, San Paolo Hospital, Department of Health Sciences, Università degli Studi di Milano, Via A. Di Rudinì n.8, 20142 Milan, Italy; 2 Clinical Epidemiology and Medical Statistics Unit, Department of Medical, Surgical and Experimental Medicine, University of Sassari, Sassari, Italy

**Keywords:** Endosonography, EBUS-TBNA, Cryobiopsy, Mediastinal lymph nodes, Lung cancer, Lymphoma, Sarcoidosis, Tuberculosis

## Abstract

Etiological diagnosis of mediastinal lymphadenopathy represents a daily challenge. Endosonography (transesophageal and transbronchial ultrasound-guided needle aspiration) is the recommended technique in the first diagnostic work-up and in the mediastinal staging of lung cancer. Despite a good sensitivity, limited amount of collected tissue may hamper molecular assessment in advanced lung cancer and in the diagnosis of lymphoproliferative disorders, fibrotic sarcoidosis, and mycobacterial lymphadenitis. Cryobiopsy, a bronchoscopic technique based on cooling, crystallization, and subsequent collection of tissue, has been successfully employed in the diagnosis of interstitial lung diseases. Cryoprobes provide larger amount of tissue than conventional bronchoscopic sampling tools and might potentially prevent the need for invasive surgical procedures. New applications of the technique (e.g., bronchoscopic diagnosis of peripheral pulmonary lesions and mediastinal lymph nodes) have been recently described in few reports. In a recent issue of the Journal, Genova et al. described five patients who underwent endobronchial ultrasound transbronchial needle aspiration (EBUS-TBNA) followed by ultrasound-guided transbronchial cryobiopsy of mediastinal lymphadenopathy for a suspected malignancy. The authors discussed about the potential added value of mediastinal cryobiopsy on a correct histopathological and molecular assessment in patients with malignancies. EBUS-cryobiopsy could be a promising technique in the diagnostic pathway of mediastinal lymphadenitis. However, cryobiopsy is now available only in few selected centres. The learning curve of the technique adapted to mediastinal ultrasound-guided sampling, the optimal sampling strategy, its true diagnostic accuracy in patients with malignant and benign diseases, as well as its safety, are still largely unclear. Mediastinal cryobiopsy could be complementary rather than alternative to conventional endosonography. Rapid on-site evaluation of EBUS-TBNA could guide subsequent sampling with cryoprobes in case of poor collection of biological material or in case of suspected lymphoproliferative disorders. Further studies should investigate its diagnostic yield, in comparison or in combination with conventional endosonography, in large cohorts of patients with malignant or benign mediastinal lymphadenopthies.

## Background

Etiological diagnosis of mediastinal lymphadenopathy represents a daily challenge [1, 2].

Thoracic and extra-thoracic malignancies or benign (i.e., sarcoidosis and mycobacterial lymphadenitis) disorders may affect the mediastinum without any pulmonary involvement and specific radiological features [1, 2].

Collection of an adequate quantity of biological specimens for cytological, molecular, and microbiological analyses is key to perform an accurate diagnosis and, then, to prescribe an appropriate therapy [1, 2].

Endosonography is the first minimally invasive technique adopted in the diagnostic work-up of mediastinal lymphadenopathies [1, 2].

An increased diagnostic accuracy can be achieved when endobronchial ultrasound transbronchial needle aspiration (EBUS-TBNA) is combined with transesophageal endoscopic ultrasound-guided fine needle aspiration (EUS-FNA) [1, 3]: it has become the gold standard in the diagnosis and mediastinal staging of non-small cell lung cancer (NSCLC) [1, 3].

However, limited amount of collected tissue may affect the molecular assessment of advanced lung cancer in about 5% of the cases [4] and hamper the diagnosis of fibrotic sarcoidosis and mycobacterial lymphadenitis [1, 5–7]. Cytological samples retrieved by needle aspiration are not sufficient for a precise histopathological characterization of mediastinal hematological malignancies in up to 33% of the cases [7] and more invasive surgical biopsies are needed.

Different needle sizes and/or aspiration techniques poorly impact the diagnostic accuracy [5]. Forceps intra-nodal biopsy shows a higher complication rate [8].

Cryobiopsy, a bronchoscopic technique based on cooling, crystallization, and subsequent collection of tissue, has recently represented a breakthrough in the diagnosis of interstitial lung diseases (ILD) [9]. Cryoprobes can provide larger amount of tissue bypassing the risk of a surgical approach [9].

On this basis, new indications (e.g., diagnosis of peripheral pulmonary lesions and mediastinal lymph nodes) have been recently described [6, 9].

## Main text

In a recent issue of the Journal Genova et al. described five patients who underwent EBUS-TBNA without rapid on-site evaluation (ROSE) followed by ultrasound-guided transbronchial cryobiopsy of mediastinal lymphadenopathy which required a histopathological assessment for diagnosis, staging and/or molecular characterization [6]. Both techniques were performed sequentially during the same endoscopic procedure, under deep sedation with propofol and midazolam [6].

EBUS-TBNA consisted of three needle passes for each selected lymph node station using a 19-gauge needle. Subsequently, two ultrasound-guided transbronchial cryobiopsies with the 1.1 mm cryoprobe were performed in one of the previously sampled lymph node station freezing, for 4 s [6]. No complications were recorded.

In two patients EBUS-TBNA findings (normal lymph node cells) were concordant with those of cryobiopsy, whereas in one patient EBUS-TBNA diagnosed a small cell lung cancer which was missed by cryobiopsy [6].

Interestingly, two cases were associated with a higher accuracy of cryoprobes in the diagnosis of malignant lymphadenopathies and this changed the management of the patients. Cryobiopsy allowed a histopathological characterization of a diffuse B cell lymphoma in a patient, and provided tissue for a histological diagnosis (squamous cell lung cancer) and subsequent molecular characterization in the other patient [6].

Another case-series described the adoption of the same technique supported by ROSE: smaller needles (i.e., 21 and 22 G) and small orifices were adequate to pass the cryoprobe into the mediastinum [10].

The authors reported successful sampling with larger biopsy specimens than EBUS-TBNA, without complications, in all patients. Cryobiopsy allowed a histopathological diagnosis in one case when suboptimal tissue samples were collected with the conventional technique.

More recently, a randomized controlled trial compared EBUS-cryobiopsy with conventional EBUS-TBNA in 197 patients with mediastinal lymphadenopathies [11]. Cryobiopsy and EBUS-TBNA were sequentially evaluated in a randomized order without ROSE. Cryobiopsy showed an overall higher diagnostic yield (91.8% vs. 79.9%, *p* = 0.001) than EBUS-TBNA. The accuracy was similar in patients with metastatic lung cancer but a higher sensitivity of cryobiopsy was detected in the subgroup of patients with uncommon malignancies (e.g. lymphoma, seminoma, thymic carcinoma) (91.7% vs. 25.0%, *p* = 0.001) and with benign lesions (i.e. sarcoidosis and tuberculosis) (80.9% vs. 53.2%, *p* = 0.004). Interestingly, in advanced NSCLC, the material collected with cryobiopsy was more frequently suitable for genotyping (93.3% vs. 73.5%, *p* < 0.001) [11].

The order of sampling methods did not affect the accuracy and no major clinical complications were recorded. However, procedural time was slightly longer and a more complex sampling technique was described. Access to mediastinal lymph nodes for the cryoprobe was created by a high-frequency needle knife, not routinely employed in diagnostic bronchoscopy [11].

EBUS-cryobiopsy represents a promising technique: it could overcome the main limitations (e.g., quantity of collected material) of needle sampling [6, 10, 11].

Several questions on its potential implementation might be raised.

Wider adoption of this technique is limited by the following multiple factors. Cryobiopsy is currently available in few selected centers. The learning curve of the technique adapted to mediastinal ultrasound-guided sampling, the optimal sampling strategy (e.g., number of passes, optimal freezing time), and the safety profile are unclear or unknown; for instance, severe bleeding in the central airways and mediastinal infections, rarely described during conventional endosonography, could be serious complications of the technique.

Furthermore, its accuracy in patients with lymphoproliferative and benign disorders should be evaluated in larger cohorts.

Mediastinal ultrasound-guided transbronchial cryobiopsy could be considered complementary, rather than alternative, to conventional endosonography. EBUS-TBNA with ROSE represents the first approach to the mediastinum; an immediate cytological assessment of the needle aspirates could guide a subsequent sampling with cryoprobes in case of poor specimen material or suspicion of lymphoproliferative disorders. Likewise, in case of necrosis and/or granulomas without evidence of malignant cells, large cryobiopsies might be collected for nucleic acid amplification tests, culture, and drug susceptibility testing.

## Conclusions

Emerging literature suggests that the use of cryobiopsy in the diagnosis of mediastinal lymphadenopathy is associated with greater diagnostic yield than conventional endosonography.

Further studies based on larger cohorts should investigate the diagnostic yield in combination with conventional endosonography both in diagnosis and staging of malignancies and benign diseases. Standardization of the sampling strategy and more detailed information on the safety profile are needed to better understand the added value in mediastinal sampling.

## Data Availability

Not applicable.

## Abbreviations

EBUS-TBNA: Endobronchial ultrasound transbronchial needle aspiration
EUS-FNA: Endoscopic ultrasound-guided fine needle aspiration
NSCLC: Non-small cell lung cancer
ROSE: Rapid on-site evaluation
ILD: Interstitial lung diseases
